# Mebendazole disrupts stromal desmoplasia and tumorigenesis in two models of pancreatic cancer

**DOI:** 10.18632/oncotarget.28014

**Published:** 2021-07-06

**Authors:** Tara Williamson, Michelle Carvalho de Abreu, Dimitri G. Trembath, Cory Brayton, Byunghak Kang, Thais Biude Mendes, Paulo Pimentel de Assumpção, Janete M. Cerutti, Gregory J. Riggins

**Affiliations:** ^1^Department of Neurosurgery, Johns Hopkins University, Baltimore, MD, USA; ^2^Oncology Research Center, Federal University of Pará, Belém, Brazil; ^3^Department of Pathology and Laboratory Medicine, University of North Carolina Hospitals, Women and Children Hospital, Chapel Hill, NC, USA; ^4^Department of Molecular and Comparative Pathobiology, Johns Hopkins University, Baltimore, MD, USA; ^5^Genetic Basis of Thyroid Tumors Laboratory, Division of Genetics, Universidade Federal de São Paulo, São Paulo, Brazil

**Keywords:** mebendazole, pancreatic cancer, cancer prevention, metastasis, mouse models

## Abstract

The five-year survival rate for metastatic pancreatic cancer is currently only 3%, which increases to 13% with local invasion only and to 39% with localized disease at diagnosis. Here we evaluated repurposed mebendazole, an approved anthelminthic drug, to determine how mebendazole might work at the different stages of pancreatic cancer formation and progression. We asked if mebendazole could prevent initiation of pancreatic intraepithelial neoplasia precursor lesions, interfere with stromal desmoplasia, or suppress tumor growth and liver metastasis. In both the *Kras*^LSL.G12D/+^; *Pdx1*-Cre (KC) mouse model of caerulein-induced inflammatory pancreatitis and the *Kras*^LSL.G12D/+^; *Tp53*^R172H/+^; *Pdx1*-Cre (KPC) mouse model of advanced pancreatic cancer, mebendazole significantly reduced pancreas weight, dysplasia and intraepithelial neoplasia formation, compared to controls. Mebendazole significantly reduced trichrome-positive fibrotic connective tissue and α-SMA-positive activated pancreatic stellate cells that heralds fibrogenesis. In the aggressive KPC model, mebendazole significantly suppressed pancreatic tumor growth, both as an early and late intervention. Mebendazole reduced the overall incidence of pancreatic cancer and severity of liver metastasis in KPC mice. Using early models of pancreatic cancer, treatment with mebendazole resulted in less inflammation, decreased dysplasia, with the later stage model additionally showing a decreased tumor burden, less advanced tumors, and a reduction of metastasis. We conclude that mebendazole should be investigated further as a component of adjuvant therapy to slow progression and prevent metastasis, and well as for primary prevention in the highest risk patients.

## INTRODUCTION

Pancreatic ductal adenocarcinoma (PDAC) is the third leading cause of cancer death in the United States [1]. The current 5-year survival rate is approximately 10%, due to its aggressive biology, early metastatic dissemination, and resistance to current therapies [2]. During microenvironmental stress associated with chronic inflammation, pancreatic acinar cells differentiate into a ductal morphology; a process known as acinar-to-ductal metaplasia (ADM) [3, 4]. The first genetic change is oncogenic activation of *KRAS,* resulting in metaplastic acinar cells undergoing terminal reprogramming [3, 5]. This process underlies the onset of low-grade pancreatic intraepithelial neoplasia (PanIN), the most common precursor of pancreatic cancer [6]. PanIN lesions can progress from low-grade (PanIN-1-2) to high-grade (PanIN-3), representing carcinoma *in situ* [7].

The evolution of pancreatic cancer pre-cursor lesions is also accompanied by a strong fibro-inflammatory desmoplastic reaction [7]. Desmoplasia results in pro-tumorigenic remodeling of the tumor stroma, fibrosis and activation of pancreatic stellate cells (PSCs), which promote tumor progression by secretion of growth factors and cytokines [8, 9]. Stromal desmoplasia is a major facilitator of PDAC progression that promotes oncogenic pathway activation [10–12]. Chronic inflammation can lead to pancreatitis, accelerate stromal desmoplasia and increase the risk of developing PDAC by as much as 16-fold [13]. The desmoplastic stroma actively contributes to tumor formation, progression, invasion, and metastasis of pancreatic cancer [8].

Mebendazole is an FDA-approved anthelminthic benzimidazole that has preclinical evidence of anticancer mechanism and activity, progressing to early-stage clinical trials. A survival benefit in a variety of malignant animal models has been observed, including melanoma [14], lung [15, 16], colorectal [17], brain [18–20], meningioma [21], breast cancer [22] and thyroid cancer [23]. Evidence suggests mebendazole exerts anticancer activity through a combination of tubulin and kinase inhibition [15, 18]. The selective binding of mebendazole in cancer cells to tubulin prevents its polymerization and results in G2/M mitotic arrest and activation of Bcl-2 and caspase-3 dependent apoptosis at nanomolar concentrations [15, 24, 25]. Mebendazole also acts as a traditional multi-tyrosine kinase inhibitor with targets that include VEGFR2, TNIK and BRAF [14, 19, 26, 27]. Inhibition of VEGFR2 pathway signaling by mebendazole leads to reduced tumor neo-angiogenesis and reduction of pro-inflammatory cytokines in colon cancer [17].

Early clinical trials using mebendazole in glioblastoma, recurrent pediatric brain tumors and colon cancer are currently underway, but sufficiently powered clinical trials have not been performed to determine if there is a survival benefit in patients with late-stage cancer.

The emphasis of translational research has been to improve therapeutics for advanced cancers. This may be at the expense of developing early-stage intervention where a greater increase in survival and quality of life could in theory be achieved. Currently, there is a relative lack of effective and low toxicity long duration therapies to use for adjuvant therapy or primary cancer chemoprevention, compared to late-stage disease. Previously, we observed that mebendazole in combination with anti-inflammatories could dramatically reduce tumor initiation and early stage growth in colon cancer models [17], and now ask if we can prevent tumor initiation, slow progression and/or prevent metastasis in mouse models of pancreatic cancer.

In this study, we determined the *in vivo* efficacy of mebendazole as a single agent therapy in two well-characterized transgenic mouse models of pancreatic cancer, the KC (*Kras*^G12D^), caerulein-induced model of PanIN progression and the KPC (*Kras*^G12D^/*Tp53*^R172H^) transgenic mouse model of advanced, metastatic PDAC. The goal of this work was to determine the timing and conditions under which mebendazole may reduce initiation and pre-neoplastic progression of early PanIN lesions, desmoplasia and incidence of PDAC in preclinical models of pancreatic cancer.

## RESULTS

### Mebendazole suppressed *Kras*-mediated, caerulein-induced tumorigenesis in the KC mouse model of pancreatitis

**Figure 1:**
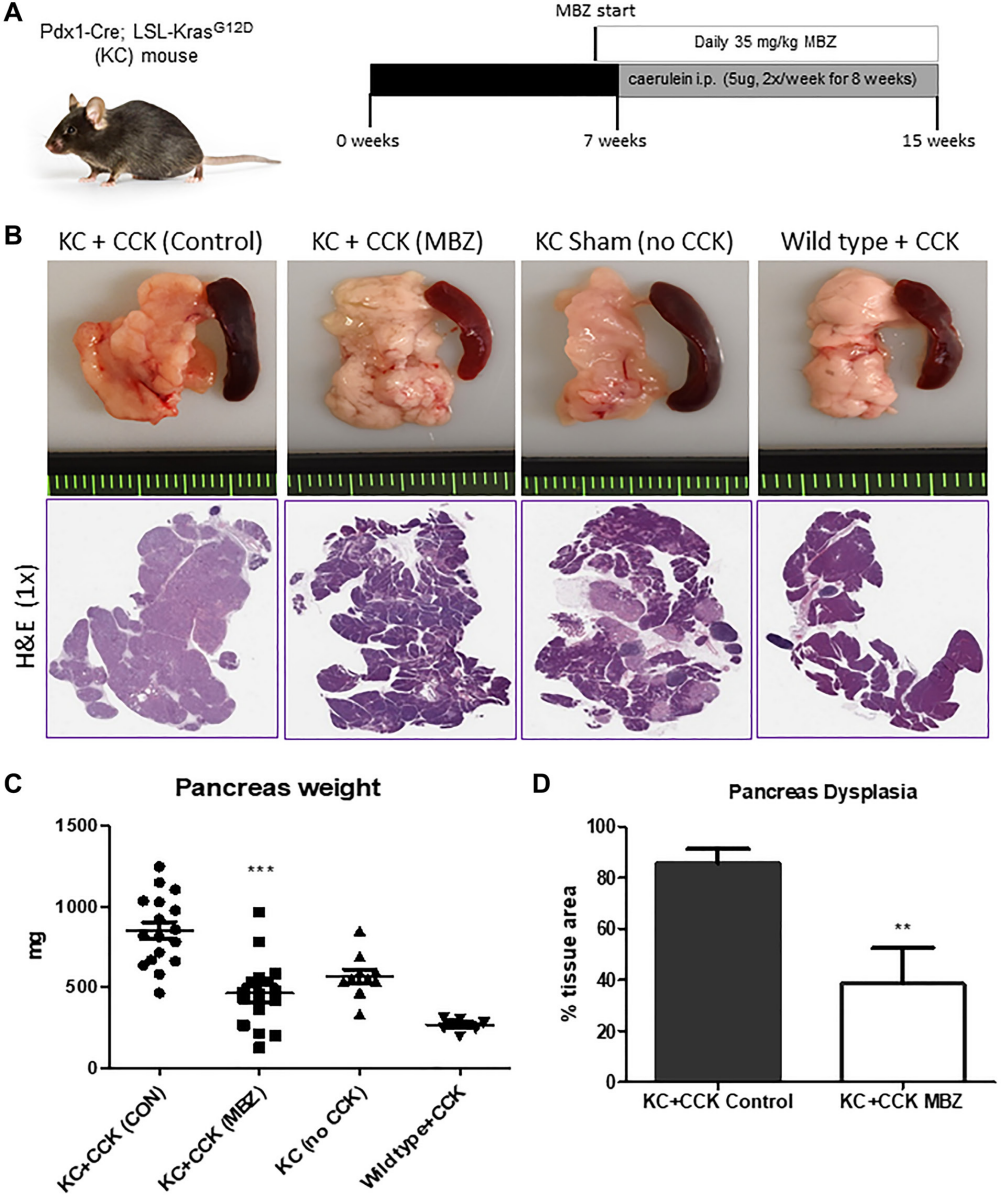
Mebendazole suppressed Kras-mediated, cearulein-induced tumorigenesis in the KC mouse model of pancreatitis. Treatment scheme and caerulein (CCK) induction schedule for KC mouse experiment (**A**). Representative pancreas and spleen tissue and H&E (1X) of untreated KC+CCK mice (*n* = 17), mebendazole (MBZ) treated KC+CCK mice (*n* = 16), Sham (no CCK, *n* = 10) KC mice and Wild type +CCK mice (*n* = 8) (**B**). Pancreas weights were compared between each treatment group (**C**). Average percent area dysplasia was calculated for untreated controls and mebendazole (MBZ) treated KC+CCK mice (**D**). Data represents mean ± S.D. *P* values ^**^ <0.01, ^***^ <0.0001.

The caerulein-induced KC mouse model is useful for studying how cancer prevention therapies can slow PanIN progression and reduce the pro-carcinogenic remodeling of the pancreas stroma under conditions of inflammatory pancreatitis. Figure 1A shows the timing of CCK induction and treatment with mebendazole in KC mice. Figure 1B displays representative pancreas and spleen tissues for untreated KC+CCK mice, mebendazole-treated KC+CCK, Sham (no CCK) KC mice and wild type mice + CCK and corresponding histology. Increased pancreas weight is a surrogate marker for progressive dysplasia and fibrosis and mebendazole treated mice had smaller, less dense pancreas tissue than untreated control mice, with H&E stained tissue sections revealing fewer areas of PanIN development following mebendazole treatment. Mebendazole in the mouse diet resulted in significantly reduced pancreas weight (851.5 mg versus 461.3 mg, *P* < 0.0001) compared to untreated KC+CCK control mice (Figure 1C). Sham-treated KC mice (PBS only, no CCK) were used as our negative control for pancreatitis. These mice still develop early PanIN lesions, which are not stimulated to progress in the absence of CCK. Mebendazole (KC+CCK) mice pancreas weights were similar in size to the Sham (no CCK) mice. Wild type mice induced with CCK displayed normal pancreas weight and morphology and did not develop PanINs or fibrosis, highlighting the need for a cooperating Kras^G12D^ mutation to induce acinar cell trans-differentiation. Sham-treated KC mice had an average pancreas weight of 570 mg whereas an average wild type mouse pancreas weighs 265.4 mg. Percent total area of dysplasia was reduced in mebendazole-treated KC+CCK mice versus untreated KC+CCK controls (85.5% versus 38.5%, *p* = 0.0066) (Figure 1D).

### Mebendazole inhibited PanIN formation and stromal desmoplasia under conditions of inflammatory pancreatitis

**Figure 2:**
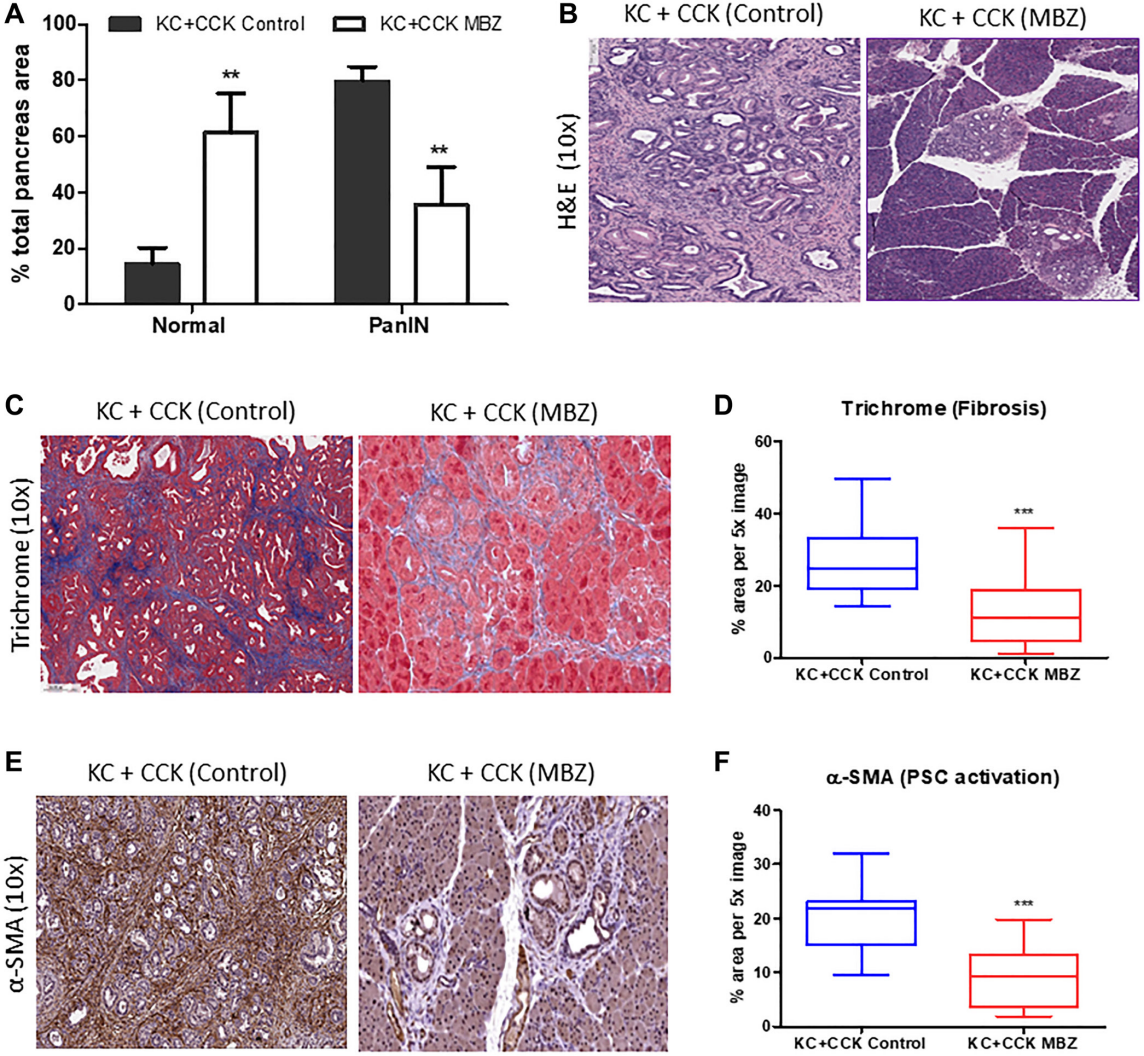
Mebendazole inhibited PanIN formation and stromal desmoplasia under conditions of inflammatory pancreatitis. Histopathological comparison of the total percentage of pancreas tissue comprised of normal acinar and PanIN (all grades) between untreated KC+CCK and mebendazole (MBZ) treated KC+CCK mice (**A**) and representative scanned H&E sections (10x) (**B**). Formalin fixed, paraffin-embedded pancreatic tissue sections from untreated control KC+CCK and MBZ-treated KC+CCK mice were analyzed for markers of stromal desmoplasia. Representative sections stained for fibrosis marker, Masson Trichrome (blue) were analyzed (10x) (**C**) and graphed for comparison (**D**). Representative sections stained for activated pancreatic stellate cell marker, α-SMA (brown) were analyzed (10x) (**E**) and graphed for comparison (**F**). Data represents mean ± S.D. *P* values ^**^ <0.01, ^***^ <0.0001.

Mebendazole-treated and untreated KC+CCK mouse pancreas tissues were further analyzed to determine the extent of PanIN formation and stromal desmoplasia. Normal acinar tissue accounted for an average of 14.4% of total tissue area in the untreated KC+CCK group versus 61.4% in mebendazole treated KC+CCK group (*p* = 0.0066). Areas of PanIN lesions (all grades) accounted for 79.7% of total tissue area in untreated mouse pancreas tissue versus 35.8% in mebendazole treated pancreas tissue (*p* = 0.0069) (Figure 2A). Representative H&E sections are shown (Figure 2B). Formalin fixed pancreas tissue sections were stained for Masson’s trichrome and α-SMA to characterize the extent of fibrotic desmoplasia and activation of pancreatic stellate cells that occurs during progression of early to late PanIN lesions. The average percent area of Masson’s Trichrome immunoreactive connective tissue was reduced in mebendazole treated pancreas tissue versus untreated controls (26.6% versus 12.6%, *p* < 0.0001) (Figure 2C and 2D). Mebendazole treated pancreas tissue also exhibited reduced areas of α-SMA immunoreactivity versus untreated controls (19.35% versus 9.38%, *p* < 0.0001) (Figure 2E and 2F). This data suggests that mebendazole is interfering with acinar-ductal trans-differentiation to PanIN lesions and stromal remodeling of the pancreas tissue in the presence of mutant-Kras and chronic inflammation.

### Mebendazole suppressed pancreatic tumor incidence and progression in the double *Kras*^G12D^/*Tp53*^R172H^ mutant KPC mouse both as an early and late intervention agent

**Figure 3:**
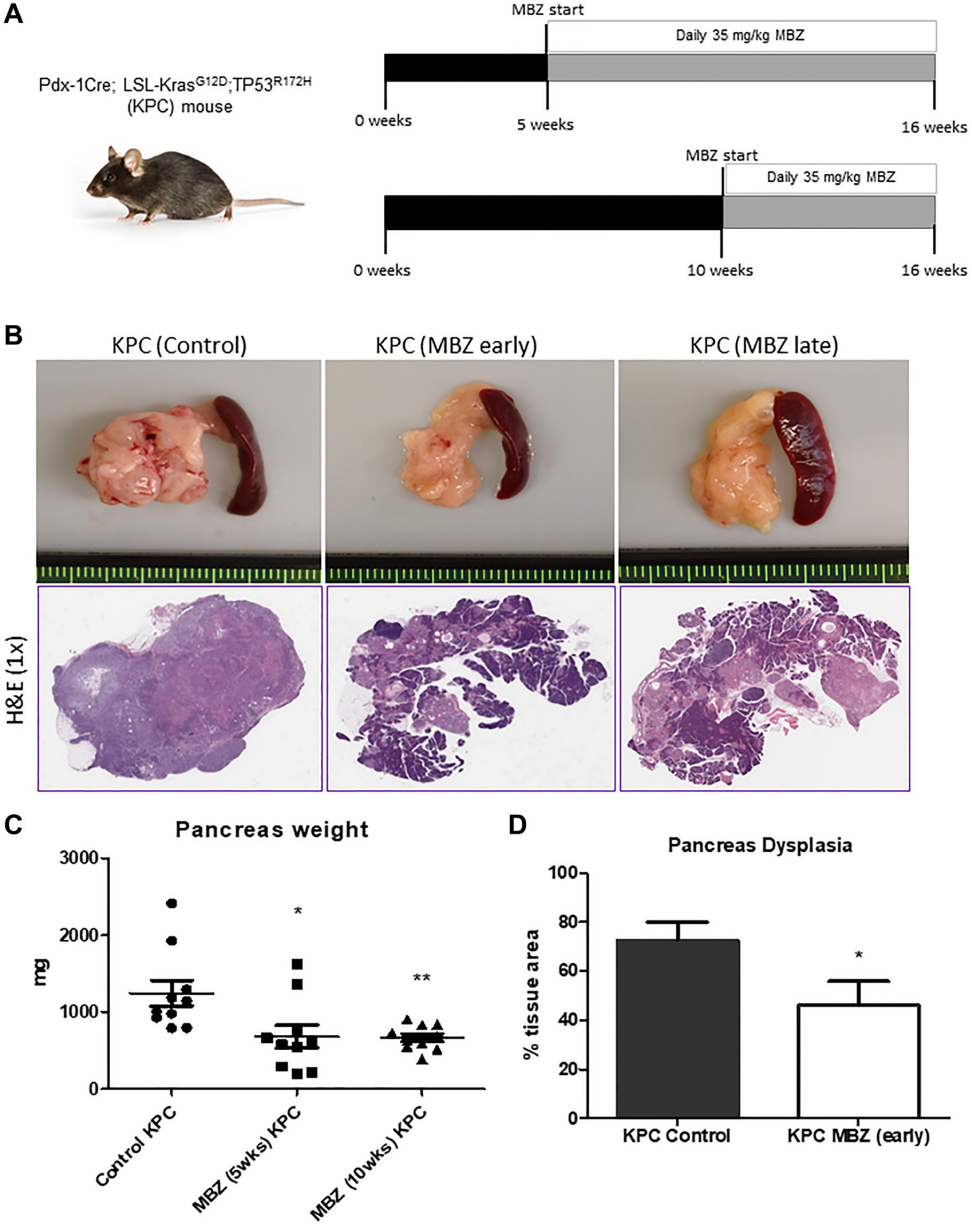
Mebendazole suppressed pancreatic tumor progression and incidence in the KPC mouse as an early and late intervention agent. Treatment scheme for KPC mouse experiments (**A**). Representative pancreas and spleen tissue and H&E (1x) of untreated control KPC, mebendazole (MBZ) early intervention KPC and MBZ late intervention KPC mice at 16 weeks of age (**B**). Pancreas weights were compared between each treatment groups (*n* = 10 mice/group) (**C**). Average percent area dysplasia was calculated for untreated controls and early treatment MBZ mice (**D**). Data represents mean ± S.D. *P* values ^*^ <0.05, ^**^ <0.01.

To test the ability of mebendazole to prevent both early PanIN initiation and progression to pancreatic ductal adenocarcinoma (PDAC), we performed an early cancer prevention and a late intervention study using KPC mice (Figure 3A). At 16 weeks of age, the pancreas tissue was harvested and analyzed. At this timepoint, some untreated KPC mice were beginning to develop clinical manifestations of late stage PDAC, such as abdominal distension and hemorrhagic ascites [28]. KPC mice that received daily oral mebendazole averaged much smaller, less dense pancreas tissue than untreated control mice, with H&E-stained tissue sections revealing fewer areas of PanIN and PDAC development in mebendazole treated mice (Figure 3B). Analysis of the collected tissues revealed that early intervention with mebendazole reduced pancreas weight (1244 mg vs. 679.3 mg, *p* = 0.0204) and spleen weight (453.1 mg vs. 210.1 mg, *p* = 0.0486) versus untreated controls. Late intervention with mebendazole reduced pancreas weight (1244 mg vs. 664.9, *P* = 0.0037) and spleen weight (453.1 mg vs. 209.8 mg, *p* = 0.0520) versus untreated controls (Figure 3C). Histopathological analysis revealed that the percent total area of dysplasia in the pancreas was reduced in mebendazole treated KPC mice versus KPC untreated controls (72.5% versus 46.1%, *p* = 0.0440) (Figure 3D). This data suggests that mebendazole is limiting tumor progression in the KPC mouse.

### Mebendazole reduced areas of PanIN and PDAC formation and stromal desmoplasia in an aggressive model of pancreatic cancer

**Figure 4:**
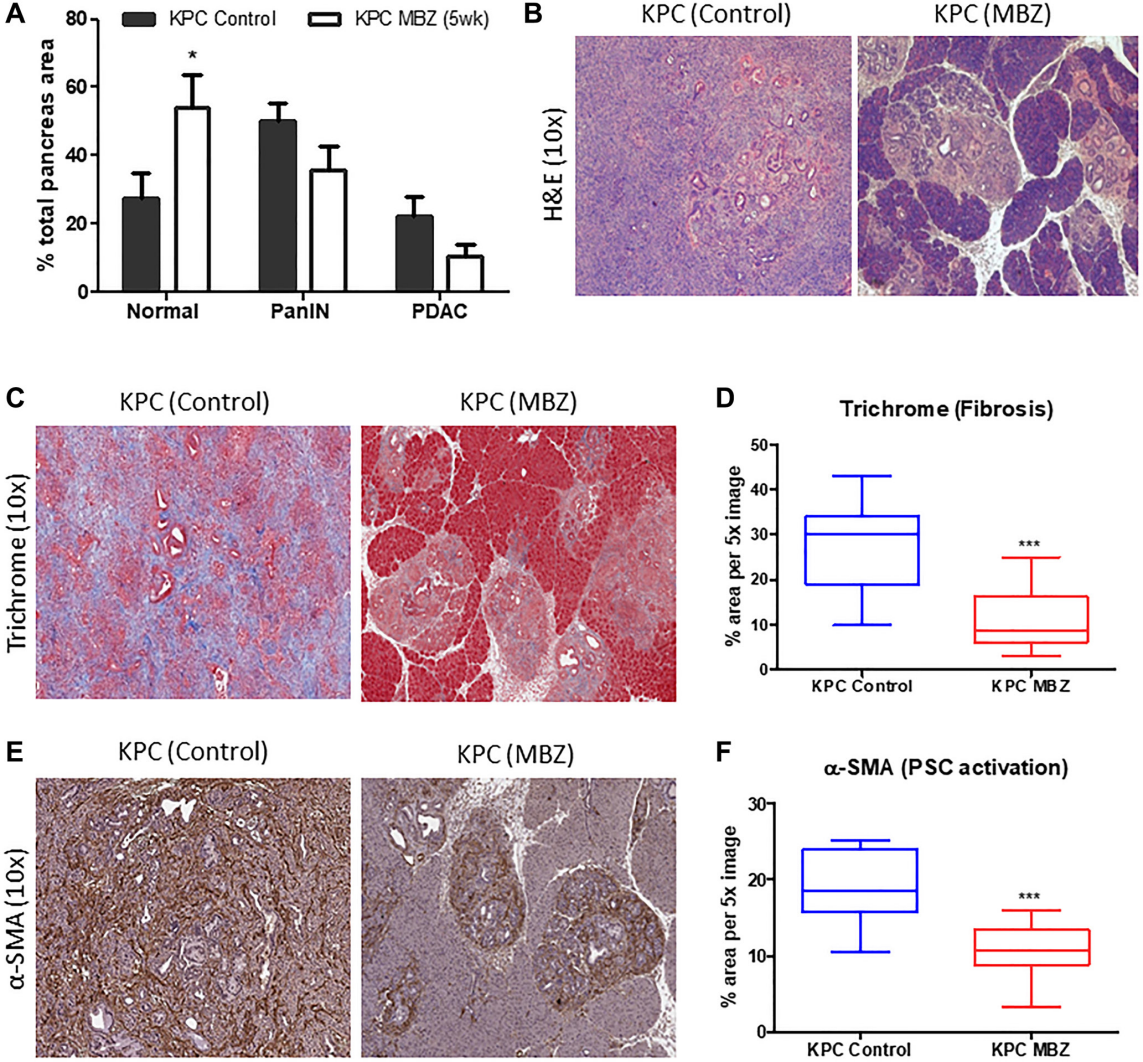
Mebendazole reduced areas of PanIN and PDAC formation and stromal desmoplasia in an aggressive model of pancreatic cancer. Histopathological comparison of the total percentage of pancreas tissue comprised of normal acinar, PanIN (all grades) and PDAC between untreated and mebendazole (MBZ) (early invention) treated KPC mice (A) and representative scanned H&E sections (10x) (B). Formalin fixed, paraffin-embedded pancreatic tissue sections from untreated and mebendazole-treated KPC mice were analyzed for markers of stromal desmoplasia. Representative sections stained for fibrosis marker, Masson Trichrome (blue) were analyzed (10x) (**C**) and graphed for comparison (**D**). Representative sections stained for activated pancreatic stellate cell marker, α-SMA (brown) were analyzed (10x) (**E**) and graphed for comparison (**F**). Data represents mean ± S.D. *P* values ^*^ <0.05, ^**^ <0.01.

Normal acinar tissue accounted for an average of 27.5% of total tissue area in untreated KPC mouse pancreas tissue versus 53.9% in mebendazole treated KPC mouse pancreas tissue (*p* = 0.0440). Areas of PanIN lesions (all grades) accounted for 50.4% of total tissue area in untreated mouse pancreas tissue versus 35.8% in mebendazole treated pancreas tissue (ns, *p* = 0.1077). Areas of PDAC formation accounted for an average of 22.2% of total tissue area in untreated mouse pancreas tissue versus 10.3% in Mebendazole treated pancreas tissue (ns, *p* = 0.1045) (Figure 4A). Representative H&E pancreas tissue sections are shown (Figure 4B). Although mebendazole may have reduced areas of PanIN and PDAC in many of the mice, the heterogenous nature of PDAC development in the KPC mouse and intragroup variability led to wide standard deviations and non-significant results. Although, the potentially more important result of PDAC incidence suggested mebendazole was slowing tumor progression at some stage in the KPC model. Mebendazole-treated (early intervention) mice exhibited a PDAC incidence of only 20% while 100% of untreated KPC mice developed PDAC tumors. Masson’s trichrome and α-SMA staining of formalin fixed pancreas tissue sections (early mebendazole intervention group) were performed to characterize the extent of fibrotic desmoplasia and activation of pancreatic stellate cells during progression to PDAC. The average percent area of Masson’s Trichrome immunoreactive connective tissue was reduced in mebendazole treated pancreas tissue versus untreated controls (27.2% versus 11.0%, *p* < 0.0001) (Figure 4C and 4D). The mebendazole treated pancreas tissue also exhibited reduced areas of α-SMA immunoreactivity versus untreated controls (19% versus 10%, *p* = 0.0001) (Figure 4E and 4F). This data indicates that mebendazole is delaying cancer progression in the KPC mouse model.

### Mebendazole reduced the severity and incidence of liver metastasis in KPC mice when administered as an early intervention

**Figure 5:**
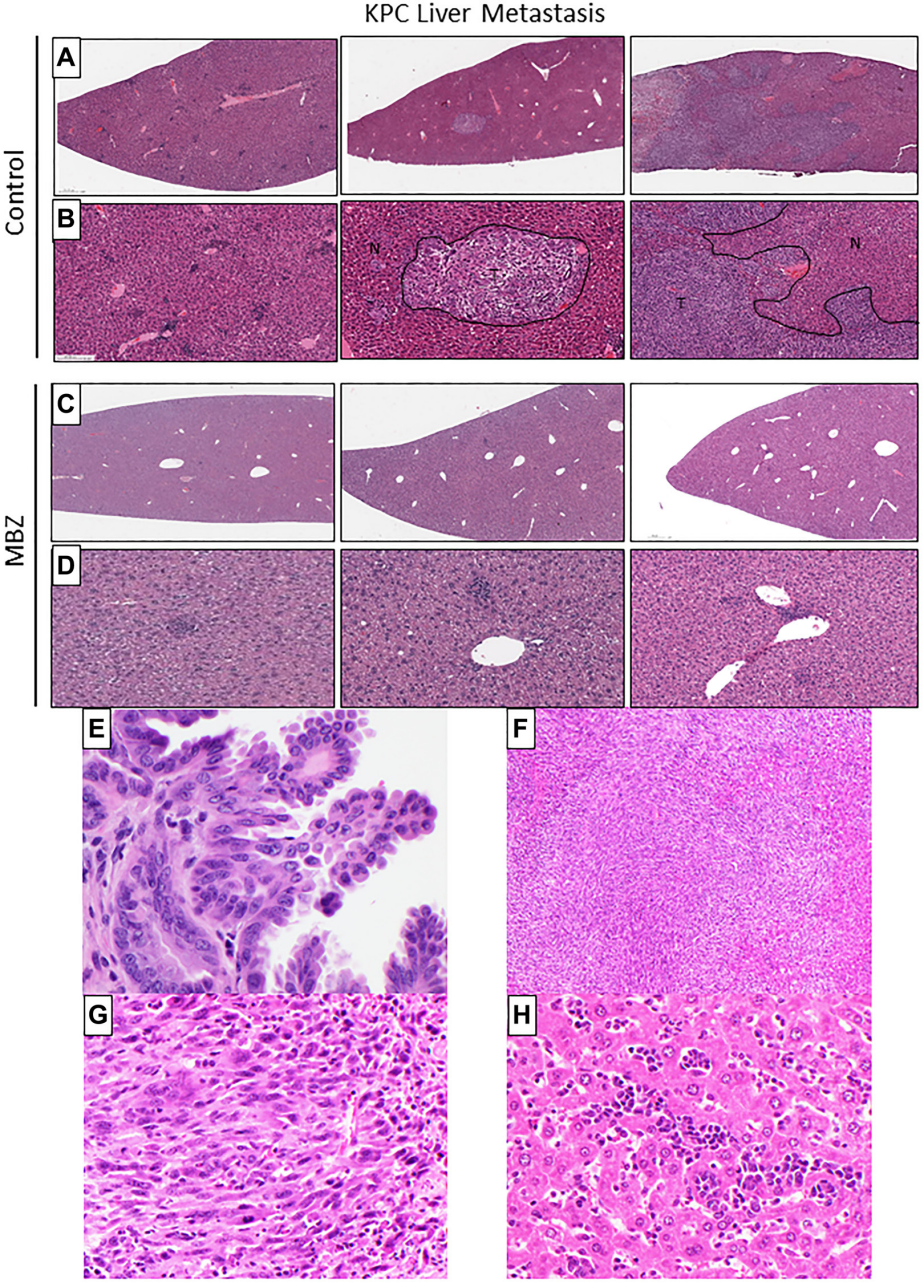
Mebendazole reduced the severity and incidence of liver metastasis in KPC mice when administered as an early intervention. Histologic evaluation of H&E stained liver tissue from 16-week old untreated KPC and early-intervention mebendazole (MBZ) treated KPC mice to compare metastasis (**A**–**D**). N = normal liver tissue. T = invasive metastatic carcinoma. Five of 5 control mice developed metastatic invasive adenocarcinomas compared to 1/5 of the treated mice. (**E**) Biliary intraepithelial neoplasia at 60x in KPC control mouse liver. (**F**, **G**) Invasive metastatic carcinoma in KPC control, 10x & 40x. (**H**) Hepatitis in KPC control, 40X.

Mebendazole has been previously shown to inhibit cancer metastasis, *in vivo* [23]. KPC control mice (*n* = 5) were compared to KPC mice treated with MBZ (*n* = 5) by a pathologist. Five of the 5 control mice developed metastatic invasive adenocarcinomas compared to 1/5 of the treated mice. Tumors in the control group ranged from moderately to poorly differentiated adenocarcinomas accompanied by moderate to severe degrees of desmoplasia (see Figure 5). Four of the 5 control animals also demonstrated biliary intraepithelial neoplasia (BilIN) ranging from low (BilIN 1, BilIN 2) to high grade (BilIN-3; see Figure 5). Only low-grade dysplasia was present in mice treated with MBZ (3/5). Interestingly, control mice showed a significant background hepatitis (see Figure 5) that was not present in mice treated with MBZ. No association was seen between degree of hepatitis and presence/absence of tumor infiltrating lymphocytes (TILs). These data are consistent with a reduced number of malignant cells in the primary tumor(s) and a greatly reduced number of malignant cells that successfully metastasized and grew.

## DISCUSSION

Currently pancreatic cancer is the third leading cause of cancer deaths in the USA but may be second by 2030 [29]. There is a projected more than two-fold increase in the number of PDAC cases in the U.S. within the next ten years [2]. Currently, only 10–20% of PDAC patients are diagnosed at an early stage allowing them to undergo the potentially curative surgical resection [30]. However, recurrence rates are high, as radical surgery has only been shown to improve the 5-year survival rate to a 20–25% [30]. The remaining 80–90% of PDAC patients present with locally advanced or metastatic disease at the time of diagnosis, about half of which are metastatic [30]. Current conventional cytotoxic treatments, multidrug regimens and targeted therapies have been mostly unsuccessful in improving long-term patient survival, offering marginal benefit with significant treatment related toxicity.

There are identifiable risk factors and markers for pancreatic cancer. Environmental risk factors such as alcohol, diet and smoking with the latter raising the risk 2- to 3-fold alone [31]. There is a high risk from inheriting certain gene mutations and one example is hereditary pancreatitis with a lifetime penetrance of 40%. Approximately 1–2% of pancreatic cysts are precancerous and analysis of the cyst fluid or other means may identify the patients most in need of early intervention including chemoprevention [32]. Mucinous lesions are common incidental findings that raise risk and are challenging, because surgery is frequently high risk and guidelines for surveillance and surgery are poorly defined.

A diagnosis of pancreatic cancer has a median life expectancy of 14 weeks, although earlier detection with circulating tumor DNA [33, 34] coupled with advances in early surgery may improve survival somewhat in the coming years. We predict that improvements in adjuvant therapy, those that prevent or reduce metastasis, a herald of end-stage disease, would be needed to be used in combination with advances in early detection, and surgery to have a substantial impact.

Given its long-standing track record of safety and previously demonstrated anti-cancer properties including colon cancer prevention [17] and prevention of metastasis in thyroid cancer [23], we sought to determine if mebendazole could reduce pre-neoplastic progression of early PanIN lesions and incidence of PDAC in preclinical models of pancreatic cancer. The efficacy of mebendazole was tested in two well-characterized transgenic mouse models of pancreatic cancer, the KC, caerulein-induced model of PanIN progression and the KPC transgenic mouse model of advanced, metastatic PDAC. Our data reveals that mebendazole-treated mice displayed reduced early *Kras*-mediated tumorigenesis, PanIN precursor formation and reduced markers of stromal desmoplasia in both models. In the aggressive KPC mouse model, mebendazole treatment reduced pancreatic tumor size and incidence and inhibited liver metastasis. Mebendazole has evidence that its long term use reduces chronic inflammation, [17] and our histopathological findings in the KC caerulein-induced pancreatitis model are consistent, but not proof, that reduction of chronic inflammation is at least in part an underlying mechanism here. This has implications for patients with chronic pancreatitis at high risk for developing PDAC. Interestingly, late intervention with mebendazole worked equally as well as early intervention mebendazole in the KPC mouse. Our data further suggests that mebendazole may also be inhibiting the early stages of PDAC tumorigenesis, leading to smaller tumors and overall, significantly less metastasis.

Mebendazole is known to prevent the polymerization of tubulin, the molecular target of the widely used anticancer drugs paclitaxel and vincristine and induce mitotic arrest selectively in tumor cells without serious adverse side effects. Mebendazole also acts as a multi kinase inhibitor [14, 19, 26, 27]. Although it is difficult to determine which molecular mechanism or combination of mechanisms contribute to the suppression of cancer initiation, progression, or metastasis; candidates include the disruption of microtubules and/or multi-tyrosine kinase inhibition. Either or both molecular mechanisms could result in one or more downstream anti-cancer effects such as increased tumor cell apoptosis, inhibiting Hedgehog or growth factor signaling between PDAC and PSC cells, and/or suppressing effectors of the RAS, PI3K/Akt or JAK/STAT3 pathways.

Mebendazole’s low toxicity profile, even at doses higher for longer duration than that used in this study, make it a candidate for long term use for chemoprevention or adjuvant therapy. Periodic blood counts and serum chemistry should be employed in this instance to screen against anemia or elevated liver enzymes, the main possible side effects. Mebendazole’s low toxicity is a further advantage in allowing mebendazole to be safely combined with other anti-cancer therapies, and possibly in combination with radiation [21, 22]. The evidence thus far suggests the possibility that survival benefit in pancreatic cancer could be enhanced by combining mebendazole with an additional treatment, and further experiments in this direction are needed.

We envision the use of a mebendazole, a safe, non-toxic drug, as an adjuvant therapy following surgical resection to prevent recurrence, or in combination with radiotherapy, targeted chemotherapy or immunotherapy to increase the durability of treatment responses in PDAC, without added toxicity. Mebendazole may have utility as an adjuvant therapy to prevent tumor recurrence in the 15–20% of PDAC patients who undergo surgery and/or to increase the durability of response to standard chemotherapy in the remaining 80–85% of patients with advanced disease. Additionally, if mebendazole alone or in combination showed promise therapeutically for advanced PDAC, it may be useful to study if morbidity and mortality can be decreased by omitting surgery for high-risk patients and/or more complex surgeries such as pancreatoduodenectomies.

Although more challenging to demonstrate in a clinical trial, mebendazole may have promise as a chemoprevention either alone or in combination with anti-inflammatories in those patients at high risk for developing pancreatic cancer, including mucinous lesions and certain inherited cancer syndromes that account for 5–10% of PDAC [35–37]. A starting point for future studies is to generate additional evidence and to optimize mebendazole for use as an adjuvant therapy to prevent metastasis and slow tumor progression after surgery in patients diagnosed with non-metastatic pancreatic cancer. Such trials are feasible with fewer patients than a primary patient trial, have a favorable benefit risk ratio, and can quickly determine if mebendazole is effective at possibly the last stage where more than a modest survival benefit can be realized.

## MATERIALS AND METHODS

### Mouse models

All experimental procedures were approved by the Johns Hopkins University Animal Care and Use Committee. The program of animal care and use is accredited by AAALAC international and follows the Guide for the Care and Use of Laboratory Animals [38]. *Kras*^LSL.G12D/+^; *Tp53*^R172H/+^ (KP) and *Pdx1*-Cre (Cre) breeding trios were a kind gift from Dr. Elizabeth Jaffee. These founders were used to create breeders for KC and KPC mouse colonies using the following crosses: A colony of KC mice were created by crossing KP (*Kras*+/−;*Tp53*+/−) females to Cre (+/+) males and a colony of KPC mice were created by crossing KP (*Kras*+/−;*Tp53*+/+;Cre−/−) males to Cre (+/+) females. Mutation status of offspring was confirmed from tail snips using Transnetyx Inc (Cordova, TN). Mice were randomized into experimental groups and males and females were used at approximately equal ratios.

### *Kras*^LSL.G12D^ *Pdx1*-Cre (KC) mouse pancreatitis study

Activation of *Kras*^G12D^ in the adult pancreas leads to non-progressing, low-grade lesion formation [39]. Chronic pancreatitis is a risk factor for malignancy and can result in the development of high-grade lesions, inflammation and desmoplasia that can progress to pancreatic ductal adenocarcinoma (PDAC) [3]. *Kras*^LSL.G12D^ *Pdx1*-Cre (KC) mice carry a *Kras*^G12D^ mutation and progressively develop acinar-to-ductal metaplasia along with low- and high-grade PanIN lesions and stromal desmoplasia, a process that is accelerated with repeated intraperitoneal injections of the proinflammatory peptide caerulein (CCK, a.k.a ceruletide) which induces pancreatitis [40, 41]. This model has been used for studying how cancer prevention therapies can slow PanIN progression and reduce the pro-carcinogenic remodeling of the pancreas stroma [42]. In our study, chronic pancreatitis was induced in 7-week old KC mice using caerulein (5 ug twice/week for 8 weeks) [43]. The control group received AIN-93G purified pelleted diet (Teklad Diet TD.94045 Envigo, Indianapolis, IN) *ad libitum*. The mebendazole group averaged a daily oral dose of 35 mg/kg mebendazole (polymorph C) achieved by mixing mebendazole with the control diet and sesame oil to enhance absorption [18]. Therapy began two days before the first CCK injection and continued throughout the duration of the study. A separate cohort of KC mice received sham injections (PBS only, no CCK) and control diet. A group of wild type C57BL6 mice received CCK using the same induction regimen listed above (5 ug, 2/week for 8 weeks) and control feed. At the end of the study, mouse pancreas and spleens were collected and weighed at the time of euthanasia and immersion fixed in 10% neutral buffered formalin for later analysis.

### *Kras*^LSL.G12D^; *Tp53*^R172H^ (KPC) mouse cancer prevention study

The KPC mouse, *Kras*^LSL.G12D/+^;*Tp53*^R172H/+^;*Pdx1*-Cre has both a *Kras*^G12D^ and a *Tp53*^R172H^ mutation in the pancreas that allows it to develop aggressive PDAC with metastatic behavior and local invasiveness that accurately mimic the human disease [40, 41]. Conditional expression of the tumor-associated *Tp53*^R172H^ mutation accelerates *Kras*^G12D^ pancreatic tumorigenesis [40, 41]. These double mutant mice develop the complete spectrum of pre-invasive PanIN, as well as end-stage, metastatic pancreatic cancer with 100% penetrance. KPC mice manifest tumors with dense desmoplasia that lead to heterogeneous advanced pancreatic ductal adenocarcinoma [44]. With a life span of approximately 5 months, chemoprevention studies can be conducted in KPC mice to study drug effects on the progression from PanINs to invasive pancreatic cancer. Histologically, five-week-old KPC mice present with mostly normal pancreas histology with a few low grade PanIN lesions, similar to KC mice. A more progressive disease is observed in animals by 10 weeks of age, when the full spectrum of pre-invasive lesions is apparent. Between 16–20 weeks of age, most KPC mice have developed locally invasive PDAC that is accompanied by a dense desmoplastic reaction [45]. In our study, KPC mice were randomized into two separate experiments: i) a fixed-point early intervention study; ii) a fixed-point late intervention study. In the early intervention study, treated mice began receiving daily 35 mg/kg mebendazole (polymorph C) in the feed starting at 5 weeks of age. In the late intervention study, treated mice began receiving daily 35 mg/kg mebendazole in the feed starting at 10 weeks of age. All untreated control mice were on untreated mouse meal (AIN-93G). Sesame oil and water (4:1 ratio) were added to all mouse chow to sufficiently mix and aid with the absorption of mebendazole. All mice were sacrificed and analyzed at 16 weeks of age. For each experiment, mouse pancreas, livers and spleens were harvested and weighed at the time of euthanasia. All tissue was placed in formalin for later analysis.

### Tissue preparation and histopathology

Formalin fixed, paraffin-embedded pancreas and liver tissues were sectioned at 5 μm spaced 50 μm apart, stained with Hematoxylin & Eosin (H&E) and representative histologic sections were scanned at 20x using an Aperio AT2 slide scanner for analysis. Comparative histopathology of pancreas sections was performed by board-certified pathologists (CB, BK) to determine the degree of dysplasia, PanIN and PDAC in H&E stained pancreas tissue sections using QuPath digital pathology image analysis software [46]. Liver H&E sections from the KPC mouse study were evaluated for degree of desmoplasia and presence of metastatic invasive adenocarcinomas and biliary intraepithelial neoplasia by a board-certified pathologist (D.T.).

### Trichrome staining and immunohistochemistry

Masson’s trichrome staining was performed on pancreas tissue sections using a Trichrome Stain kit from Abcam, according to the manufacturer’s protocol. For immunohistochemistry, paraffin-embedded pancreas tissue slides were deparaffinized with xylene and hydrated through a graded alcohol series. Endogenous peroxidase activity was blocked with 3% hydrogen peroxide (Fisher Scientific) for 10 min. Antigen retrieval was performed using Antigen Retrieval Citra Solution pH 6 (BioGenex) in a heat cooker for 30 min, followed by avidin/biotin block (Life Technologies) with 10% goat serum + 1% BSA in phosphate-buffered saline and incubated for 30 min. Primary antibody for alpha-smooth muscle actin (α-*SMA,* 1:200, Abcam) was incubated overnight. Immunostaining was performed using Super Sensitive^™^ IHC Detection Systems (BioGenex). Slides were counterstained with DAB chromagen. Both Masson’s trichrome and α-SMA stained slides were scanned at 20x using an Aperio AT2 slide scanner. Three fields were randomly selected from five pancreas tissue slides in each group, and the percent positively stained area in each field was determined using Image J software.

### Statistical analysis

The data are presented as the mean ± SD. Comparisons between groups were analyzed by Student’s *t*-test using GraphPad Prism software (GraphPad Prism version 6.0; La Jolla, CA, USA). Kaplan-Meier analysis was used for survival analysis. Statistical significance was declared based on a *p*-value <0.05.
